# Synthesis of Starch-Grafted Polymethyl Methacrylate via Free Radical Polymerization Reaction and Its Application for the Uptake of Methylene Blue

**DOI:** 10.3390/molecules27185844

**Published:** 2022-09-09

**Authors:** Uzma Yasmeen, Fazal Haq, Mehwish Kiran, Arshad Farid, Naveed Ullah, Tariq Aziz, Muhammad Haroon, Sahid Mehmood, Muhammad Muzammal, Shakira Ghazanfar, Majid Alhomrani, Abdulhakeem S. Alamri, Syed Mohammed Basheeruddin Asdaq, Saleh A. Alghamdi, Irfan Ullah

**Affiliations:** 1 Institute of Chemical Sciences, Gomal University, Dera Ismail Khan 29050, Pakistan; 2 Department of Horticulture, Faculty of Agriculture, Gomal University, Dera Ismail Khan 29050, Pakistan; 3 Gomal Center of Biochemistry and Biotechnology, Gomal University, Dera Ismail Khan 29050, Pakistan; 4 School of Engineering, Westlake University, Hangzhou 310024, China; 5 Department of Chemistry, University of Turbat, Balochistan 92600, Pakistan; 6 State Key Laboratory, Zhejiang University, Hangzhou 310027, China; 7 National Institute of Genomics and Advanced Biotechnology (NIGAB), National Agricultural Research Centre, Park Road, Islamabad 45500, Pakistan; 8 Department of Clinical Laboratory Sciences, The Faculty of Applied Medical Sciences, Taif University, Taif 21944, Saudi Arabia; 9 Centre of Biomedical Sciences Research (CBSR), Deanship of Scientific Research, Taif University, Taif 21944, Saudi Arabia; 10 Department of Pharmacy Practice, College of Pharmacy, AlMaarefa University, Dariyah, Riyadh 13713, Saudi Arabia or; 11 Medical Genetics, Clinical Laboratory Department, College of Applied Medical Sciences, Taif University, Taif 21944, Saudi Arabia; 12 Department of Life Sciences, School of Science, University of Management and Technology (UMT), C-II Johar Town, Lahore 54770, Pakistan

**Keywords:** St-g-PMMA, grafting, adsorption, methylene blue, kinetics, isotherms

## Abstract

In this research, a new biodegradable and eco-friendly adsorbent, starch-grafted polymethyl methacrylate (St-g-PMMA) was synthesized. The St-g-PMMA was synthesized by a free radical polymerization reaction in which methyl methacrylate (MMA) was grafted onto a starch polymer chain. The reaction was performed in water in the presence of a potassium persulfate (KPS) initiator. The structure and different properties of the St-g-PMMA was explored by FT-IR, 1H NMR, TGA, SEM and XRD. After characterization, the St-g-PMMA was used for the removal of MB dye. Different adsorption parameters, such as effect of adsorbent dose, effect of pH, effect of initial concentration of dye solution, effect of contact time and comparative adsorption study were investigated. The St-g-PMMA showed a maximum removal percentage (R%) of 97% towards MB. The other parameters, such as the isothermal and kinetic models, were fitted to the experimental data. The results showed that the Langmuir adsorption and pseudo second order kinetic models were best fitted to experimental data with a regression coefficient of R^2^ = 0.93 and 0.99, respectively.

## 1. Introduction

Dyes are commonly used in the manufacturing of cosmetics, textiles, paper, plastic and food products [1,2]. Most of these dyes are drained directly into fresh water and can make it damaging to human health and unfit for aquatic life [3,4]. Methylene blue is a basic cationic dye, usually used to color paper, silk, leathers and cotton [5,6]. It causes various types of diseases in human beings, such as hepatic problems, skin irritation, allergies and problems in the central nervous system [7]. Therefore, these dyes must be eliminated from wastewater in order to reduce their negative impact on aquatic and terrestrial life [8,9]. To date, a variety of methods, including membrane filtering, biological processes, chemical oxidation, adsorption, coagulation-flocculation and precipitation are employed to remove dyes [10]. Among these methods, adsorption is the most widely used method as it is more effective and affordable [11]. Similarly, different adsorbents are used for the adsorption of dyes, such as activated carbon, carbon nanotubes, carbon nanocages, zeolite, titanate nanosheets, mesoporous silica and starch. Starch is the most favored adsorbent due to its wide availability, biodegradability and efficiency. The other adsorbents mentioned above tend to be expensive, non-selective and non-biodegradable, and they may act as a secondary pollutant [12]. Starch is a biopolymer present as stored food material in plants and grains [13]. Starch has a large number of hydroxyl groups on its main chain, which can easily be replaced with different functional groups to improve its adsorption behavior towards toxic dyes. The chemically modified starches produce specific interactions, such as hydrogen bonding and electrostatic interaction, with the dye molecules [14]. The functionality of starch can be modified in different ways using physical, chemical and enzymatic techniques [15]. The chemical modification technique is one of the most favored techniques as it increases the number of functional groups on the backbone of the starch. The most vital function of the chemically modified starch is its function as an adsorbent for the removal of dyes [16].

In recent years, different materials have been tested for the removal of MB dye from wastewater. Jordana et al., used raw sawdust of Pinus Elliottii for the removal of MB dye from an aqueous solution. The raw sawdust showed a removal percentage of 97% towards the MB dye [17]. Similarly, for the uptake of MB dye, Imron et al., used Lemna Minor (an aquatic plant) as an adsorbent. The said material showed a removal efficiency of 82% towards the MB dye [18]. For the removal of MB dye, ethylenediamine tetraacetic acid (EDTA) embedded nanocellulose (NCED) was tested as a sorbent. The NCED showed a removal percentage of 91% for MB dye. All the above-mentioned materials exhibited good sorption affinity for MB dye [19].

However, the synthesis of these materials takes a lot of time, and the adsorption efficiency still needs to be improved.

In this research, we have synthesized starch-grafted polymethyl methacrylate (St-g-PMMA) via free radical polymerization reaction. The new procedure we adopted for the synthesis of St-g-PMMA reduced the synthetic time. Secondly, the St-g-PMMA was found to be an efficient adsorbent compared with the other previously described adsorbents, with a removal percentage of 97% towards MB dye. The main reason for the selection of starch-based materials for this research was that these materials are eco-friendly. Starch costs about USD 0.05 kg^−1^, which explains why starch-based biopolymers are less expensive than other biopolymers such as poly (lactic acid) (PLA), polyhydroxyalkanoate (PHA) or polyesters [20]. PMMA is also a cheaper alternative to polycarbonate (PC) in applications such as tensile strength, flexural strength, transparency, polishability and UV tolerance [21,22,23]. From an ecological point of view, starch and PMMA are both biodegradable and eco-friendly. Ming et al., showed the biodegradable nature of starch by successfully degrading the starch film via α-amylase [24]. Additionally, there are numerous examples in the literature that show the bio-degradable nature of PMMA-based materials. Thakore et al., checked the bio-degradability of PMMA-starch-cinnamate blends in different solvents. The obtained results showed that the PMMA-starch-cinnamate blends exhibited a degradation of 30% in 120 days [25]. Similar results were reported by Bhat et al., when they effectively biodegraded PMMA blends with cellulose acetate and cellulose acetate phthalate [26]. Thus, the highly adsorptive, biodegradable and environmentally friendly nature of starch-grafted polymethyl methacrylate mean that it is ideally suited for use as an adsorbent for the removal of MB dye from wastewater.

## 2. Results

For the synthesis of St-g-PMAA, MMA was grafted onto starch using the free radical polymerization technique. The structure and typical properties of the St-g-PMAAs were investigated using different techniques, the details of which are outlined below.

**Figure 1:**
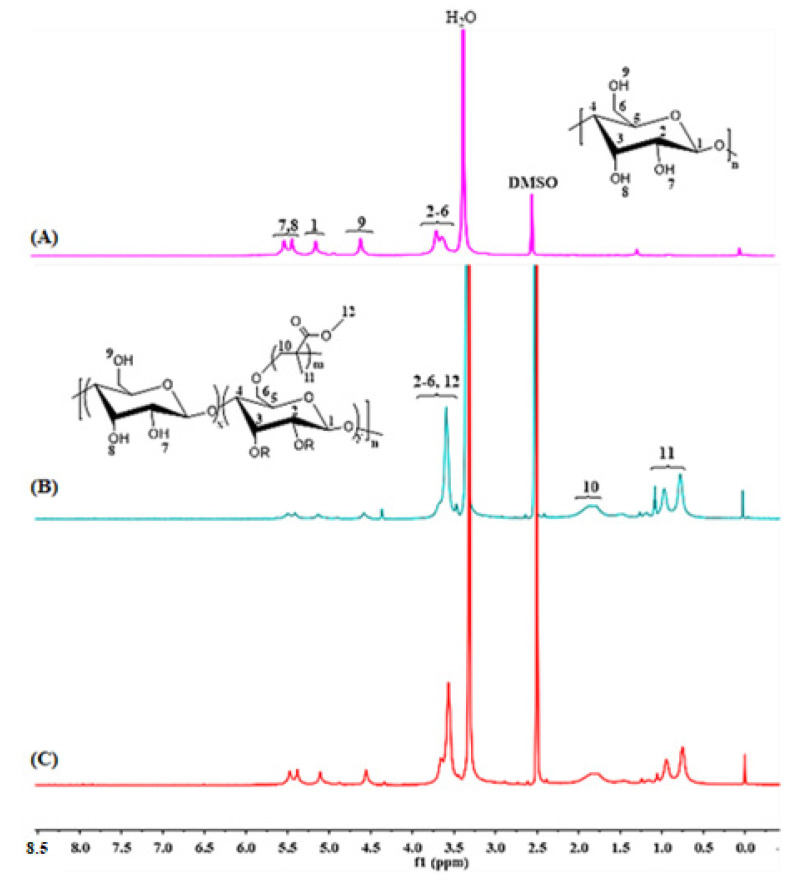
The ^1^H NMR spectra of (**A**) starch, (**B**) St-g-PMMA1 and (**C**) St-g-PMMA2.

The ^1^H NMR spectra of the starch, St-g-PMMA1 and St-g-PMMA2 are shown in Figure 1. In Figure 1A, the starch spectrum showed a peak in the range δ = 4.90–5.22 ppm, which was associated with a single proton of C1, while six protons from C2 to C6 of AGU were revealed to make up the peaks at δ = 3.56–3.81 ppm. The peak at δ = 4.40–4.68 ppm was assigned to a proton of the hydroxyl group attached to C6 (H9). Similarly, it was determined that the protons of the hydroxyl groups connected to C2 and C3 (H7, H8) were likely responsible for the peak in the region of δ = 5.33–5.53 ppm [27]. After polymerization, the new peaks (shown in Figure 1A,B) for the eight protons of C10, C11 and C12 appeared at δ = 0.73–3.81 ppm [11]. However, the signals of the C12 protons at 3.5–3.8 ppm overlapped with those of the C2 to C6 protons at δ = 3.56–3.81 ppm. The appearance of all these new peaks in the St-g-PMMA1 and St-g-PMMA2, which were absent in the starch spectra, indicated successful polymerization. Similar results were also reported by Haq et al., when they synthesized starch-grafted polymethyl methacrylate via the ATRP technique [7]. The relative grafting ratio (GRr) was calculated by applying the following Equation (1).

(1) $$\mathrm{GRr}=\frac{area\;of\;C-10\;protons\;of\;grafted\;PMMA/2}{area\;of\;C-1\;proton\;of\;starch\;main\;chain}$$

The GRr values for St-g-PMMA1 and St-g-PMMA2 were found to be 0.9 and 1.2 mol/mol, respectively. These results suggest that St-g-PMMA2 has high ratio of PMMA.

### 2.1. FT-IR Analysis

**Figure 2:**
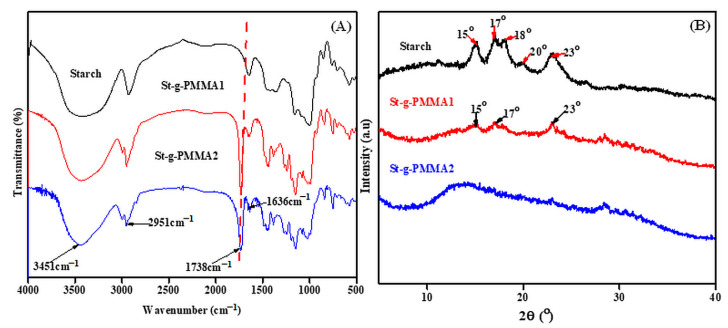
(**A**) FT-IR and (**B**) XRD studies of the starch and St-g-PMAAs.

The FT-IR analysis was undertaken to check the existence of different functional groups in the structure of the starch and St-g-PMMAs. In the spectrum of the starch, different peaks were observed at 3451 cm^−1^ (stretching frequency of -OH), 2951 cm^−1^ (-CH stretching frequency) [28], 1636 cm^−1^ (C-O stretching frequency) and 841 cm^−1^ (stretching frequency of C-O-C). Correspondingly, the above peaks were also present in the FT-IR spectra of the St-g-PMMA1 and St-g-PMMA2. However, a new peak at 1738 cm^−1^ appeared in the St-g-PMMA1 and St-g-PMMA2 spectra, which was absent in the starch spectrum [29]. This peak was due to the carbonyl group of the ester in the St-g-PMMA1 and St-g-PMMA2. The occurrence of these new peaks in the FT-IR spectra of St-g-PMMA1 and St-g-PMMA2 evidently supports the production of St-g-PMMAs. The FT-IR spectra are shown in Figure 2A.

The XRD patterns for the starch, St-g-PMMA1 and St-g-PMMA2 are shown in Figure 2B. The study demonstrated that starch has a semi-crystalline nature. Strong diffraction peaks for starch can be found at 2θ = 15°, 17°, 18°, 20° and 23° [30]. Similarly, some small diffraction peaks were found in the St-g-PMMA1 at 2θ = 15°, 18° and 23°, but it exhibited very weak crystalline peaks [8]. After the grafting of the PMMA onto the starch, the crystalline natures of both the individual components were lost, which showed the amorphous nature of the St-g-PMMAs. The splitting of intra-molecular hydrogen bonding during grafting was theorized to be the cause of the loss of crystallinity [31]. The crystalline structure of the starch and the amorphous nature of the St-g-PMMAs provide proof that these materials were successfully synthesized.

**Figure 3:**
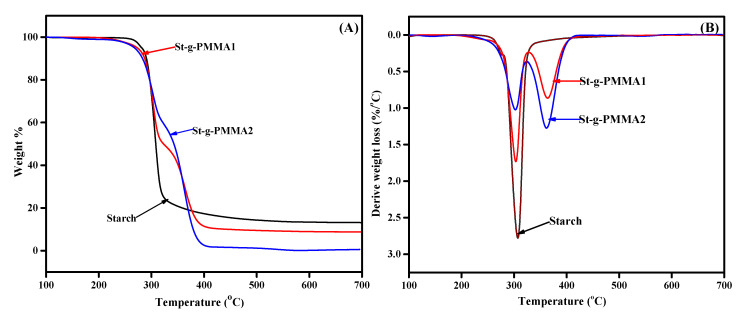
(**A**) TGA and (**B**) DTG curves of the starch and St-g-PMAAs.

The thermal behavior of the starch and St-g-PMMAs are shown in Figure 3A,B. The curve of thermogravimetric analyses for the starch showed a single-step thermal decomposition at temperature 307 °C, which was attributed to the breakdown of AGU and C-O-C glycosidic linkage [32]. The TGA and DTG curves for the St-g-PMMA1 and St-g-PMMA2 exhibited two-step thermal decomposition [33]. The first steps of the decomposition of the S-g-PMMA1 and S-g-PMMA2 occurred at 303 °C and 301 °C, respectively, and this was attributed to the interruption of the starch part. The second steps of the decomposition of the St-g-PMMA1 and St-g-PMMA2 were observed at temperature 363 °C and 361 °C, respectively. This decomposition was attributed to the collapse of the grafted PMMA portions [1]. The two stages of the thermal decomposition of the St-g-PMMAs confirmed the successful grafting of the PMMA onto the starch.

**Figure 4:**
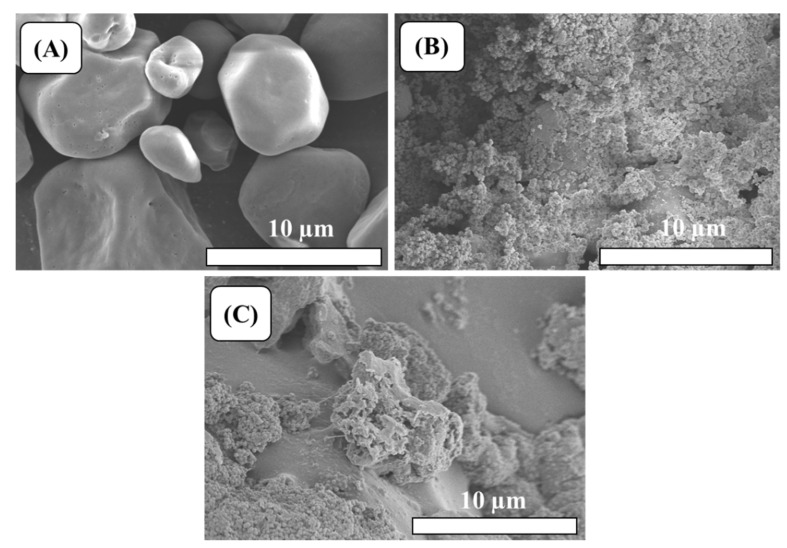
SEM photographs of (**A**) starch, (**B**) St-g-PMMA1 and (**C**) St-g-PMMA2.

SEM examination was used to examine the surface morphology of the compounds. The surface morphologies of the starch, St-g-PMMA1 and St-g-PMMA2 are shown in Figure 4A–C. The particles of starch exhibited smooth surfaces and round and polyhedral shapes [34]. After the grafting of the PMMA onto the starch, the morphologies of the S-g-PMMAs were entirely altered. The grafting of the PMMA onto the backbone of the starch was responsible for the morphological change. During the grafting, the majority of the hydrogen bonds were broken, and the starch’s compact structure distorted. The St-g-PMMA1 and St-g-PMMA2 particles showed porous and rough surfaces with greater surface area, which are supposed to be good for adsorption [35].

### 2.2. Applications

**Figure 5:**
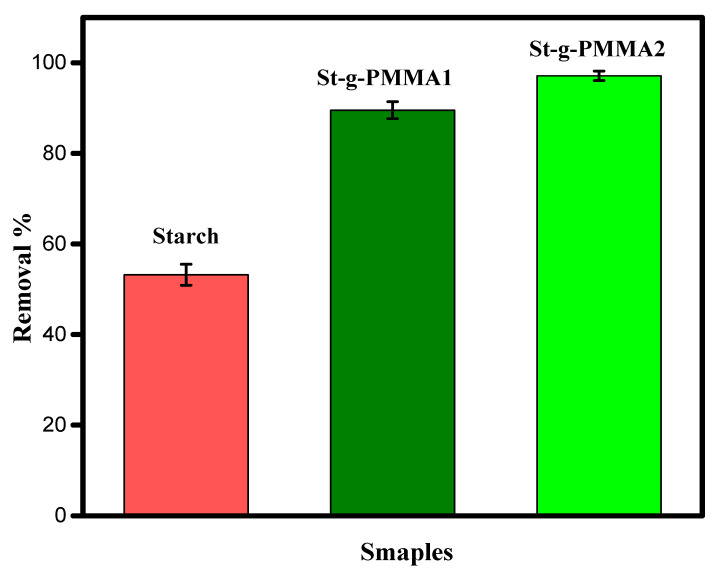
Uptake of MB by starch, St-g-PMMA1 and St-g-PMMA2.

To check the effect of grafting on adsorption, a comparative adsorption study was conducted. The obtained results for the comparative adsorption study are shown in Figure 5.

The %R of MB for the starch, S-g-PMMA1 and S-g-PMMA2 were determined to be 53.2%, 89.5% and 97.1%, respectively. The grafting of the PMMA onto the backbone of the starch was responsible for the high %R. During the synthesis of S-g-PMMA2, the molar ratio of starch to MMA was kept at 1:6. The ^1^H NMR study also showed a high relative grafting ratio for S-g-PMMA2. Thus, the high %R for S-g-PMMA2 was attributed to the high density of PMMA on the backbone of the starch. The second-highest value of %R, 89.5%, was observed for S-g-PMMA1. The S-g-PMMA1 therefore had a lower PMMA value. The Lowest %R, 53.2%, was observed for the native starch. These results show that in the starch, only the hydroxyl groups were involved in the uptake of the MB molecules, while in the S-g-PMMAs, both the hydroxyl groups and the PMMA particles were involved in the uptake of the MB molecules. These experiments show that grafting can successfully increase the adsorption capacity of starch. Similar results were obtained by Fazal et al., who used carboxymethyl starch-grafted to polyvinyl imidazole for the uptake of phenol and found that the grafting effectively increased the adsorption ability of the material [8].

#### 2.2.1. Effect of Adsorbent Dose

**Figure 6:**
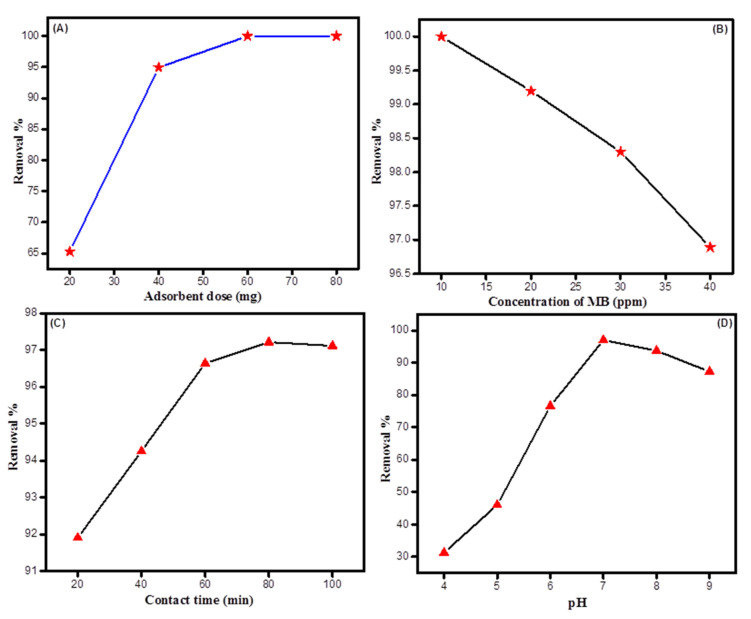
(**A**) Effect of adsorbent dose, (**B**) effect of initial concentration of dye, (**C**) effect of contact time and (**D**) effect of pH.

The adsorbent dose plays a vital role in adsorption. To check this effect, the adsorption behavior of S-g-PMMA2 towards MB was investigated at different doses (20, 40, 60 and 80 mg). The results obtained for the adsorbent dose experiment are shown in Figure 6A. The findings showed that increasing the adsorbent dose can linearly increase the %R. It was observed that an increase in adsorbent dosage from 20 to 80 mg increased the %R from 65% to 100%. This increase might be attributed to the increase in the number of active sites on the adsorbent, or, in other words, an increase in the number of hydroxyl groups and PMMA particles. As these groups were responsible for the uptake of MB, they effectively increase the %R. Similar results were reported by Omar et al., who used Fava bean peels for the removal of MB [36]. A similar effect was also observed by Runping et al., who used cereal chaff for the removal of MB dye [37].

#### 2.2.2. Effect of Initial Concentration of Dye

The initial concentration of the dye solution determines the accessibility of the dye molecules in the solution. Generally, increasing the initial concentration of dye decreases the %R because there are more dye molecules for the same number of active sites on the adsorbent surface. The results of varying the initial dye concentrations are shown in Figure 6B. The figure shows that as the initial dye concentration was increased from 10 to 20 ppm, the %R decreased from 100% to 97%. These results suggest that at 10 ppm the number of MB molecules was relatively low compared with the large number of active sites on the adsorbent. Thus, all of the dye molecules were captured by the adsorbent, and it showed an %R of 100%. However, when the initial concentration of MB was increased from 10 to 40 ppm, the %R decreased. This change might be attributed to the reduced availability of active sites on the surface of the adsorbent due to the high number of dye molecules in the solution. A similar effect was observed by Linda et al., while using Terminalia catappa shell for the removal of MB dye from wastewater [38].

#### 2.2.3. Effect of Contact Time

The results relating to the effect of contact time on the uptake of MB dye by St-g-PMMA2 is shown in Figure 6C. This figure indicates that the %R was increased from 91.9% to 97.12% by increasing the contact time from 20 min to 80 min. After 80 min, it was observed that the %R was almost constant, which indicated that by 80 min the reaction had achieved equilibrium. At this exact time, the active sites on St-g-PMMA2 were saturated by adsorbing MB molecules, resulting in a maximum %R of 97.2%. The %R did not rise significantly as the contact time was increased further, up to 100 min. These results show that after 80 min, the active sites on the St-g-PMMA2 were completely saturated and were no longer available for MB molecules. In the beginning, the rate of %R was fast because at this stage the active sites on the surface of the adsorbate were vacant and ready for MB molecules. As time passed, the rate of %R decreased as the dye molecules occupied the vacant active sites of the adsorbent. The results of our experiment are supported by the work of Deepak et al., who tested activated carbon developed from Ficus carica for the removal of MB dye [39].

#### 2.2.4. Effect of pH

The pH of the solution is one of the vital factors affecting the adsorption process [40]. The results relating to the effect of the pH on the adsorption experiments are shown in Figure 6D. We can observe from this figure that at a low pH the %R was lowered because the active sites on the adsorbent, such as the hydroxyl and ester groups, were protonated due to a high H^+^ concentration in the solution. Therefore, at this stage, there was no interaction between the adsorbent and the dye molecules. When the pH of the solution was increased to 7.6, the highest %R (97.02%) was achieved, because at this point the hydroxyl groups along with the PMMA particles were deprotonated [41]. This deprotonation converted the surface of the adsorbent into a negatively charged bed which adsorbed the cationic dye via electrostatic interactions. By further increasing the pH, a decrease in %R was observed. At high pH, a greater number of hydroxyl groups competed with the positively charged MB molecules and retarded the free flow of MB molecules towards the negatively charged adsorbent. A similar effect was reported by Abdul et al., when they used polyacrylonitrile-based activated carbon nanofibers for the removal of MB dye from an aqueous solution [42].

### 2.3. Kinetic and Isotherm Studies

Adsorption isotherm plays a key role in the identification of adsorption mechanisms and the determination of adsorption capacity [41]. In this research, to observe the order of adsorption reactions between the St-g-PMMAs and MB dye, pseudo first order (Equation (1)) and pseudo kinetic (Equation (2)) models were applied to experimental data [43].

(2) $$\ln\left(q_{e}-q_{t}\right)=\ln q_{e}-k_{1}t\;$$

(3) $$\;\frac{t}{q_{t}}=\frac{1}{k_{2}q_{e}^{2\;}}+\frac{1}{q_{e}}t\;\;\;$$

According to Equation (2), a plot of $\ln\left(q_{t}-q_{e}\right)$ as a function of time (*t*) provides estimates of $q_{e}\;$ and $k_{1}$ from the intercept and the slope terms, respectively. Similarly, in Equation (3), a plot of ($\frac{t}{q_{t}}$) versus t provides values of $k_{2}$ and qe from the intercept and slope, respectively.

**Figure 7:**
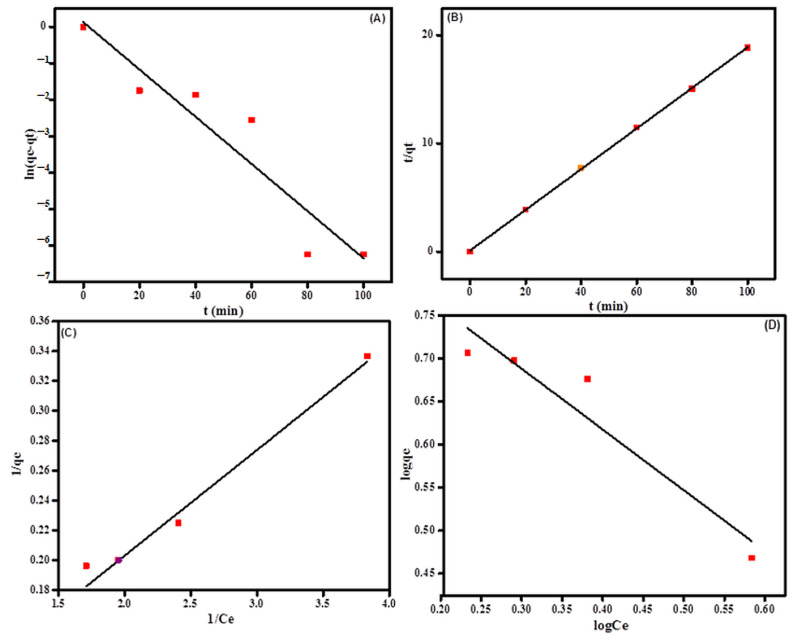
(**A**) Pseudo first order kinetic model, (**B**) pseudo second order kinetic model, (**C**) Langmuir isotherm and (**D**) Freundlich isotherm models.

Where $k_{1}$ is the pseudo first order rate constant (min^−1^), $k_{2}$ is the pseudo second order rate constant (g·mg^−1^·min^−1^), *q_e_* is the equilibrium adsorption capacity (mg/g) and *q_t_* is the adsorption capacity over time (mg/g). All of the data were collected by running the adsorption experiment under different conditions such as MB dye concentration (40 ppm), stirring rate (450 rpm), contact time (20, 40, 60, 80 and 100 min), pH (7.6) and St-g-PMMA (60 mg). The results are shown in Figure 5. This figure shows that the pseudo second order kinetic model was best fitted to the experimental data compared with the pseudo first order kinetic model, and specifies the chemisorption mechanism. The value of linear regression coefficient ($R^{2}$) for the pseudo second order model was found to be 0.99, which was higher than that of the pseudo first order kinetic model (0.86). The results for the pseudo first order model and pseudo second order model are shown in Figure 7A,B and Table 1.

In order to find the adsorption behavior between S-g-PMMAs and MB dye, Langmuir and Freundlich isotherm models were applied to the experimental data. The equilibrium data at varying concentrations of MB dye (40 ppm) were examined in this study. The other adsorption experiments were conducted under different conditions, such as S-g-PMMAs (60 mg), contact time (80 min), pH (7.6) and stirring rate (450). The Langmuir (Equation (4)) [44] and Freundlich isotherm (Equation (5)) [45] models can be written as follows:

(4) $$\frac{1}{q_{e}}=\frac{1}{K_{L}q_{max}}\times \frac{1}{C_{e}}+\frac{1}{q_{max}}$$

(5) $$\log q_{e}=\log K_{f}+\frac{1}{n}\log C_{e}\;\;\;\;\;\;\;\;\;\;\;\;\;\;\;\;$$

The amount of dye adsorbed by the adsorbent is represented by *q_e_* (mg/g); the equilibrium dye solution concentration is represented by *C_e_* (mg/L); the maximal monolayer adsorption capacity is represented by *q_max_* (mg/g); and the Langmuir constant is represented by *k_L_* (L/mg). *K_f_* is the adsorbent capacity and *n* is the adsorption intensity constant in the Freundlich isotherm equation. According to the results illustrated in Figure 7, the Langmuir isotherm model was better for modeling the elimination of MB dye by St-g-PMMAs. The value of linear regression coefficient (R^2^) for the Langmuir’s isotherm model was observed to be 0.93, which was higher than that for the Freundlich isotherm (0.87). Thus, the Langmuir isotherm model suggested the monolayer adsorption.

**Figure 8:**
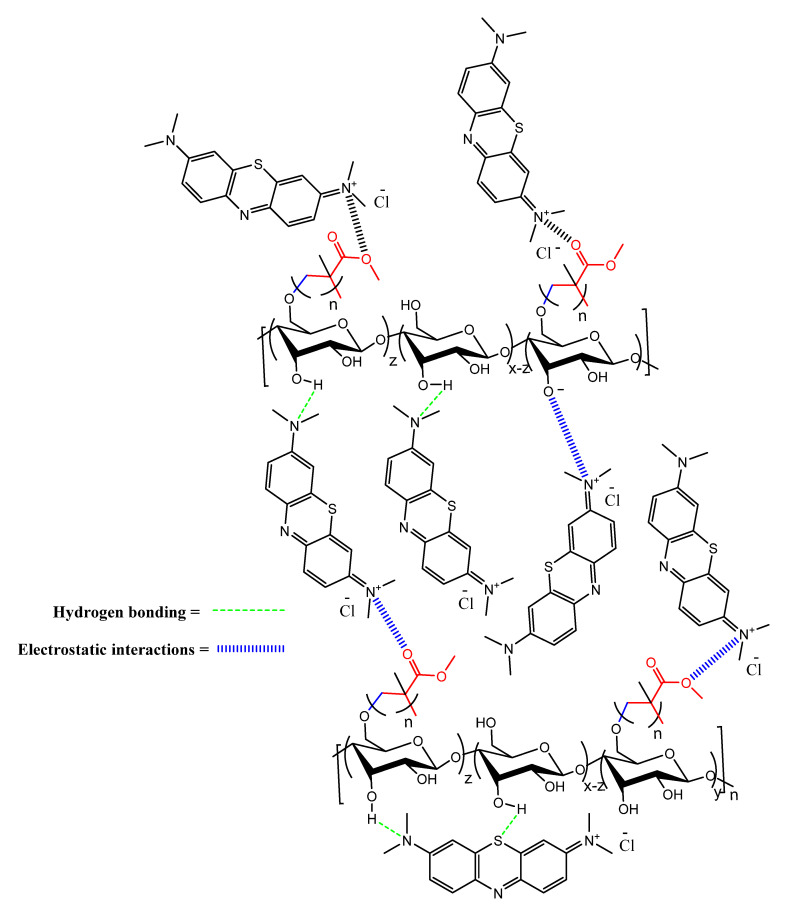
Connections between the St-g-PMMA2 and the MB dye.

The specific interactions that are produced between the adsorbent and adsorbate are described by the adsorption mechanism. The MB dye and St-g-PMMAs have generated two different forms of interactions, i.e., electrostatic interaction and hydrogen bonding. The hydrogen bonds were generated between the lone pair of electrons of the nitrogen and sulphur of the structure of the MB dye and the hydrogen of the hydroxyl group of the St-g-PMMAs [46,47]. Additionally, the MB dye’s positively charged nitrogen atom and negatively charged oxygen atoms on the starch backbone and the methyl methacrylate established electrostatic connections. At pH 7.6, electrostatic interactions were developed between the positive charge of the nitrogen in the MB and the negative charge of the oxygen of the St-g-PMMAs, since the hydroxyl groups were deprotonated at this pH. Figure 8 depicts the various interactions between the St-g-PMMAs and the MB dye.

## 3. Materials and Methods

Corn starch (purity ≥99%), sodium hydroxide (NaOH) (purity ≥98%) and hydrochloric acid (HCl) (purity 37%) were purchased from Sigma Aldrich (St. Louis, MO, USA). Methyl methacrylate (MMA) (purity ≥99%), Methylene Blue (MB), potassium persulfate (KPS) (purity ≥99%), ethanol (99.7%) and methanol (99.5%) were bought from Acros Organics (Geel, Belgium).

### 3.1. Synthesis of St-g-PMMA

**Figure 9:**
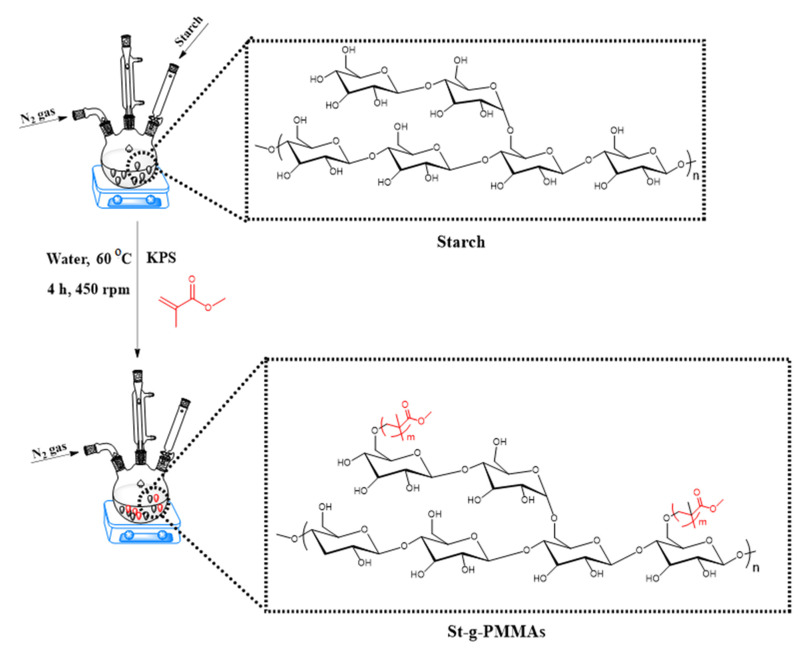
Synthesis of St-g-PMMA.

The synthesis of St-g-PMMA was carried out in a three-necked round-bottom flask in an inert atmosphere of nitrogen. First, 1 g (6.1 mmol of AGU) of starch was dispersed in 40 mL of distilled water in a three-necked round-bottom flask. The reaction was run for 30 min at a temperature of 60 °C and a stirring rate of 450 rpm. Then, in 10 mL of distilled water, 66.49 mg (24.64 mmol) of KPS was dissolved and injected into the reaction mixture. The reaction was continued for another 30 min at the same temperature and stirring rate. Next, 2 mL (18.32 mmol) of MMA was taken in a 5 mL syringe and injected into the reaction contents present in the three-necked round-bottom flask. The reaction was then maintained for an additional two hours at the same temperature and stirring rate. The reaction was stopped after two hours, and the resulting mixture was cooled. To make the reaction mixture free of homopolymers and unreacted MMA, it was washed three times with a mixture of methanol and distilled water (30:70) via centrifugation (3000 rpm for 10 min). After centrifugation the supernatant was discarded and the pellets containing the product were washed two times with ethanol to remove the methanol and water. Finally, the product was placed in a vacuum oven at a temperature of 40 °C for 24 h to completely dry. The procedure adopted for the synthesis of St-g-PMMA is shown in Figure 9 and Table S1.

### 3.2. Characterization

The details of every instrument used to characterize the starch and synthesize St-g-PMMAs are given below.

The ^1^H NMR spectra of the starch and St-g-PMMA were taken using a Bruker Advanx-400 DMX NMR spectrometer (Boston, MA, USA). For ^1^H NMR spectra, 5 mg each of St-g-PMMA1 and St-g-PMMA2 were dissolved in 10 mL of DMSO. Then, the sample solution was taken in NMR tubes and analyzed by NMR spectrometer. The FT-IR spectra of the starch and St-g-PMMAs were recorded on a Nicolet 5700 IR spectrometer (Boston, MA, USA). For the FT-IR measurement, KBr was mixed with 5% St-g-PMMA1 and St-g-PMMA2 separately, and the mixed powder was pressed using a manual hydraulic press machine, producing KBr pellets. The thermal stability of the starch and St-g-PMMAs was obtained on a thermogravimetric analyzer (TA-Q500, Mettler-Toledo, Leuvensesteenweg, Belgium). For TGA, 3 mg each of St-g-PMMA1 and St-g-PMMA2 were taken in a sample pan and placed in the thermogravimetric analyzer. During this typical process, the samples were heated at a rate of 10 °C/min from 50 °C to 700 °C under an inert atmosphere of nitrogen. The crystalline nature was measured with an X-ray diffractometer (XPert PRO, CuKα, λ = 1.54, Malvern, UK). The surface morphology of the starch and St-g-PMMAs was obtained using an SEM spectrometer (SU-8010, Atlanta, GA, USA). For the SEM study, the St-g-PMMA1 and St-g-PMMA2 were converted into fine powder by pestle and mortar. Then, a small quantity of each sample was placed on a Cu grid and analyzed by SEM spectrometer. During the experiments, the pH of the different solutions was checked by InoLab pH 7110 (Roubaix, France). During this procedure, the probe of the pH meter was dipped in the solutions of different concentrations, and the concentrations of each solution were noted from the digital display of the pH meter. A centrifuge machine (HERMLE Z 206 A, Sayreville, NJ, USA) was used to separate the unwanted materials from the product. In this process, the sample solutions were taken in centrifuge tubes and placed in the centrifuge machine at 3000 rpm for 10 min each time. The centrifuge machine separated the materials by centrifugal force. The materials of higher density settled at the bottom of the centrifuge tube. As the desired product was present in pellets, the unwanted materials present in supernatant were easily discarded. The concentration of MB dye in various solutions was determined using the IRMECO-U2020 UV/Visible spectrophotometer (Waltham, MA, USA). For this procedure, the base line was corrected by taking water as the reference solvent. After the base line correction, the concentrations of the different ppm solutions were determined and a standard curve was obtained. For the adsorption experiments, the concentration of MB dye in the supernatant was checked using a UV-Visible spectrophotometer at λ_max_ 665 nm. All the readings were taken in triplicate.

### 3.3. Adsorption Experiments

A series of adsorption experiments were conducted to examine the MB removal capability of St-g-PMMA. Initially, a 100 ppm stock solution was prepared by dissolving 100 mg of MB in 1000 mL of DW. Then, the other solutions of desired concentrations were prepared by diluting the stock solution. For the comparative adsorption experiment, 50 mg each of starch, St-g-PMMA1 and St-g-PMMA2 were mixed separately with 20 mL of MB solution (40 ppm), and the solutions were gradually stirred at 450 rpm for 80 min. Next, a UV-Visible spectrophotometer was used to determine the amount of MB dye present in the supernatant at the maximum wavelength (λmax = 666 nm). The various adsorption parameters, including the pH of the solution, adsorbent dose, contact time, initial dye concentration, kinetic models and adsorption isotherms, were examined throughout this investigation.

#### 3.3.1. Comparative Adsorption Study

The objective of the comparative study was to examine the impact of PMMA grafting on adsorption. During this study, 50 mg each of starch and the St-g-PMMAs were added individually to two 25 mL Erlenmeyer flasks containing 20 mL of MB solution of 40 ppm. The Erlenmeyer flasks were paced on heating plates at a stirring rate of 450 rpm, a temperature of 25 °C and a pH of 7.6 for 80 min. Subsequently, the solution was centrifuged at 3000 rpm and the supernatant was collected with a syringe from the centrifuge tube. The concentration of MB in the supernatant was checked using a UV-Visible spectrophotometer. The percentage removal (%R) was calculated by using Equation (6).

(6) $$\%\;R=\frac{\left(C_{i}-C_{f}\right)}{C_{i}}\times 100$$

Here, “*%R*” is the removal percentage, while $“C_{i}”$ and $“C_{f}”$ are the initial and equilibrium concentrations (mg/L) of the MB dye, respectively.

#### 3.3.2. Effect of Adsorbent Dose

One of the vital parameters that affect the adsorption process is the adsorbent dose. In order to check the effect of the adsorbent dose, four experiments were carried out, in which the amount of St-g-PMMA2 was varied from 20–80 mg while the other experimental conditions were kept constant (i.e., initial concentration of the MB solution 40 ppm, pH of the solution 7.6, stirring rate 450 rpm and contact time 80 min).

#### 3.3.3. Effect of Initial Concentration of Dye

Four experiments were conducted to determine the effect of the initial concentration of dye on adsorption. The uptake of MB by the St-g-PMMAs was tested in 20 mL MB solutions having concentrations of 10 to 40 ppm. The other experimental conditions (adsorbent dose 50 mg, pH of the solution 7.6, temperature 25 °C, stirring rate 450 rpm and contact time 80 min) were kept constant.

#### 3.3.4. Effect of Contact Time

The contact time is an important part of the adsorption process as it explains the time required for the uptake of the MB dye by the adsorbent. Five adsorption experiments at various contact times (20, 40, 60, 80 and 100 min) were carried out in this study to examine the effect of contact time on the %R. These experiments were run under the reaction conditions of pH 7.6, temperature 25 °C, adsorbent dose 50 mg, stirring rate 450 rpm and concentration of MB solution 40 ppm.

#### 3.3.5. Effect of pH

During this study, to check the effect of the pH on adsorption, the adsorption experiment was conducted at different pH values (4–9). The basic and acidic pH conditions were adjusted to the desired levels by adding HCl (0.1 M) and NaOH (0.1 M) to the solution. The adsorption experiment was performed under the conditions of adsorbent dose 50 mg, temperature 25 °C, stirring rate 450 rpm, concentration of MB solution 40 ppm and contact time 80 min.

## 4. Conclusions

The St-g-PMMAs were synthesized by using a free radical polymerization reaction. The St-g-PMMAs were characterized by spectroscopic techniques such as FT-IR and 1H NMR. The other properties were also examined using TGA, DTG, XRD and SEM. The results showed that the grafting of the PMMA onto the backbone of the starch diminished the crystalline nature of the starch, and that the St-g-PMMAs showed an amorphous nature. The grafting also increased the degradation temperature from 303 °C to 363 °C. It was further noticed that the grafting of the PMMA onto the backbone of the starch completely distorted the morphology of starch granules, and that the St-g-PMMAs showed rougher surfaces. The S-g-PMMAs were used for the removal of the MB dye from the model solution. The results showed that the grafting of the PMMA increased the removal percentage of 44% with respect to native starch. It was further discovered that increasing the adsorbent dose (20–80 mg) linearly increased the removal percentage from 65% to 100%. The St-g-PMMAs exhibited a maximum removal percentage of 97% towards the MB dye at a contact time of 80 min, pH of 7.6, initial dye concentration of 40 ppm and adsorbent dose of 60 mg. The pseudo second order kinetic model suggested that the adsorption process favored the chemisorption mechanism due to a higher linear regression coefficient value (R^2^), i.e., 0.99. The Langmuir adsorption isotherm suggested that the monolayer adsorption of the MB dye occurred on the surface of the St-g-PMMAs. On the basis of these results, we can conclude that St-g-PMMAs can be used as an adsorbent for the removal of MB dye from wastewater.

## Figures and Tables

**Table 1:** Kinetic and Isotherm parameters for the removal of MB dye by the St-g-PMMAs.

(a) Kinetic Parameters		
Order of reaction	Parameters	MB dye
Pseudo first order	q_e_ (mg/g)	1.142
	K_1_ (min^−1^)	−0.00065
	R^2^	0.86
Pseudo second order		
	q_e,cal_ (mg/g)	5.32
	K_2_ (g mg^−1^·min^−1^)	0.302
	R^2^	0.99
**(b) Isotherm Parameters**		
Types of isotherm	Parameters	MB dye
Langmuir	q_max_ (mg/g)	16.28
	K_L_ (g mg^−1^ min^−1^) R_L_	0.867 0.081
Freundlich	R^2^ K_f_ 1/n R^2^	0.92 7.95 −0.70 0.87

## Data Availability

Not applicable.

## Supplementary Materials

The following supporting information can be downloaded at: https://www.mdpi.com/article/10.3390/molecules27185844/s1, Table S1: The synthesis of various St-g-PMMAs.
