# Supplementary material for: Synthesis of Starch-Grafted Polymethyl Methacrylate via Free Radical Polymerization Reaction and Its Application for the Uptake of Methylene Blue
Source: Molecules. 2022 Sep 9;27(18):5844. doi: 10.3390/molecules27185844 (PMC9503754; doi:10.3390/molecules27185844)
Supplement: Supplementary file 1 [file molecules-27-05844-s001.zip › molecules-1885552-supplementary.pdf]

Supporting Information

Synthesis of Starch-Grafted Polymethyl Methacrylate via Free Radical Polymerization Reaction and Its Application for the Uptake of Methylene Blue

Uzma Yasmeen[1], Fazal Haq[1*], Mehwish Kiran[2], Arshad Farid[3*], Naveed Ullah[1], Tariq Aziz[4], Muhammad Haroon[5], Sahid Mehmood[6], Muhammad Muzammal[3], Shakira Ghazanfar[7], Majid Alhomrani[8,9], Abdulhakeem S. Alamri[8,9], Syed Mohammed Basheeruddin Asdaq[10], Saleh A. Alghamdi[11], Irfan Ullah1[2*]

Table S1. The synthesis of various St-g-PMMAs

Run	Sample	Step 1					Step 2					Step 3			
		Starch		Water	t_1	T_1	KPS		Water	t_2	T_2	MMA		t_3	T_3
		g	mmol (AGU)	mL	h	℃	mg	mmol	mL	h	℃	mL	mmol	h	℃
1	St-g-PMMA1	1	6.25	50	0.5	60	66.5	0.25	10	0.5	60	2	18.3	2	60
2	St-g-PMMA2	1	6.25	50	0.5	60	117	0.43	10	0.5	60	3.9	37	2	60
